# Afferent Input Selects NMDA Receptor Subtype to Determine the Persistency of Hippocampal LTP in Freely Behaving Mice

**DOI:** 10.3389/fnsyn.2016.00033

**Published:** 2016-10-21

**Authors:** Jesús J. Ballesteros, Arne Buschler, Georg Köhr, Denise Manahan-Vaughan

**Affiliations:** ^1^Department of Neurophysiology, Medical Faculty, Ruhr University BochumBochum, Germany; ^2^Max Planck Institute for Medical ResearchHeidelberg, Germany

**Keywords:** mouse, LTP, NMDA, *in vivo*, GluN2A, GluN2B, synaptic plasticity, hippocampus

## Abstract

The glutamatergic N-methyl-D-aspartate receptor (NMDAR) is critically involved in many forms of hippocampus-dependent memory that may be enabled by synaptic plasticity. Behavioral studies with NMDAR antagonists and NMDAR subunit (GluN2) mutants revealed distinct contributions from GluN2A- and GluN2B-containing NMDARs to rapidly and slowly acquired memory performance. Furthermore, studies of synaptic plasticity, in genetically modified mice *in vitro*, suggest that GluN2A and GluN2B may contribute in different ways to the induction and longevity of synaptic plasticity. In contrast to the hippocampal slice preparation, in *behaving* mice, the afferent frequencies that induce synaptic plasticity are very restricted and specific. In fact, it is the stimulus pattern and not variations in afferent frequency that determine the longevity of long-term potentiation (LTP) *in vivo*. Here, we explored the contribution of GluN2A and GluN2B to LTP of differing magnitudes and persistence in freely behaving mice. We applied differing high-frequency stimulation (HFS) patterns at 100 Hz to the hippocampal CA1 region, to induce NMDAR-dependent LTP in wild-type (WT) mice, that endured for <1 h (early (E)-LTP), (LTP, 2–4 h) or >24 h (late (L)-LTP). In GluN2A-knockout (KO) mice, E-LTP (HFS, 50 pulses) was significantly reduced in magnitude and duration, whereas LTP (HFS, 2 × 50 pulses) and L-LTP (HFS, 4 × 50 pulses) were unaffected compared to responses in WT animals. By contrast, pharmacological antagonism of GluN2B in WT had no effect on E-LTP but significantly prevented LTP. E-LTP and LTP were significantly impaired by GluN2B antagonism in GluN2A-KO mice. These data indicate that the pattern of afferent stimulation is decisive for the recruitment of distinct GluN2A and GluN2B signaling pathways that in turn determine the persistency of hippocampal LTP. Whereas brief bursts of patterned stimulation preferentially recruit GluN2A and lead to weak and short-lived forms of LTP, prolonged, more intense, afferent activation recruits GluN2B and leads to robust and persistent LTP. These unique signal-response properties of GluN2A and GluN2B enable qualitative differentiation of information encoding in hippocampal synapses.

## Introduction

The N-methyl-D-aspartate receptor (NMDAR) plays a key-role in hippocampus-dependent learning, hippocampal synaptic plasticity (Shipton and Paulsen, 2013) and memory encoding (Morris et al., 1986; for review see Morris, 2013). In fact, the NMDAR is likely to contribute to coincidence detection of neuronal activity. Thus, for the NMDAR to be effectively activated, not only must glutamate bind to the receptor, but its glycine site must be occupied (Johnson and Ascher, 1987). In addition, the voltage-dependent Mg^2+^-block must be removed from the channel pore (Mayer et al., 1984). In other words, both glutamate release and the associated membrane depolarization must be substantial and sustained in order for NMDARs to be activated. Physiologically, this can be expected to occur when information in the form of afferent impulses derived, for example, from sensory information that has been pre-processed by the entorhinal cortex (Lavenex and Amaral, 2000), and from neuromodulatory/arousal networks (Sara, 2009; Hansen and Manahan-Vaughan, 2014), converge on synapses of the hippocampus. Notably, behavioral studies with GluN2 NMDAR mutants have revealed different contributions from GluN2A- and GluN2B-containing NMDARs to memory performance (von Engelhardt et al., 2008; Cui et al., 2013; Kannangara et al., 2014). Whereas GluN2A is required for rapidly acquired spatial working memory (Bannerman et al., 2008), GluN2B is critical for a long-delay working memory task (Zhang et al., 2013).

NMDAR typically comprise two GluN1 subunits and two GluN2 subunits (Dingledine et al., 1999). The co-agonist binding site located on the GluN1 subunit, is activated by glycine or D-serine binding (Hirai et al., 1996; Mothet et al., 2000; Henneberger et al., 2010). Glutamate binds to the GluN2 subunit (McBain and Mayer, 1994; Laube et al., 1997), and the GluN2 composition of NMDARs determines the receptor kinetics. For example, GluN2A-containing NMDARs are known to have faster rise and decay times than GluN2B-containing NMDARs (e.g., Punnakkal et al., 2012), and although they have a similar affinity for Mg^2+^ (Kuner and Schoepfer, 1996), GluN2A-containing NMDARs unblock faster from Mg^2+^ (Clarke and Johnson, 2006; Clarke et al., 2013). It is widely accepted that the GluN1 subunit reflects a necessary element for synaptic plasticity in the hippocampus, and in particular the CA1 region (Tsien et al., 1996), but the specific role of the GluN2 subunits in synaptic plasticity still remains unclear. In hippocampal and cortical slices, it has been reported that antagonism of GluN2A subunits prevents long-term potentiation (LTP), whereas antagonism of GluN2B subunits leads to inhibition of long-term depression (LTD; Liu et al., 2004; Massey et al., 2004). LTD is also impaired in hippocampal slices from transgenic mice that are deficient in GluN2B (Kutsuwada et al., 1996; Brigman et al., 2010). In contrast, other *in vitro* studies have shown that GluN2B is not required for LTD (Hendricson et al., 2002; Morishita et al., 2007) and that both GluN2 subunits are involved in hippocampal LTP (Köhr et al., 2003; Berberich et al., 2005; Pawlak et al., 2005; Bartlett et al., 2007). All of the above mentioned studies were conducted using the hippocampal slice preparation and examined the effects in transgenic mice that lack either GluN2A or GluN2B. *In vitro* studies in *rats* have not made the picture clearer (Shipton and Paulsen, 2013; Volianskis et al., 2013). No *in vivo* studies of synaptic plasticity in GluN2A/GluN2B genetically modified mice have been conducted, but in anesthetized (Fox et al., 2006) or behaving rats (Manahan-Vaughan, 1997; Lemon et al., 2009), pharmacological studies suggest that both subunits are involved in LTP and LTD. Here, there are two clear confounds: pharmacological agents are never perfectly specific, and the stimulation protocols that are used to induce LTP and LTD in the mouse hippocampus *in vitro* do not generate the same result in the behaving mouse (Buschler et al., 2012; Goh and Manahan-Vaughan, 2013a). Thus, the role of GluN2A and GluN2B in hippocampal synaptic plasticity remains an unresolved controversy.

Our understanding of how hippocampal synaptic plasticity relates to learning is steadily improving: on the one hand, robust associative memory that is generated through context experience is encoded by LTP in the hippocampus (Whitlock et al., 2006). On the other hand, spatial memory requires both LTP and LTD (Kemp and Manahan-Vaughan, 2004, 2007, 2012). The specific nature of afferent stimuli converging on the hippocampus determines the potency and experience-dependent content of information encoding through synaptic plasticity (Frey et al., 2001; Lemon and Manahan-Vaughan, 2006; Lemon et al., 2009; Hagena and Manahan-Vaughan, 2011, 2015). From this perspective, it may not be the afferent frequency *per se*, but rather the *pattern* with which afferent information reaches the hippocampus that determines not only the durability, but also the precise content of synaptic plasticity and the memory it encodes.

In this study, we examined the signaling role of GluN2A- and GluN2B-containing NMDARs in synaptic plasticity of different durations that we induced with an identical afferent stimulation frequency, but different stimulus patterns in freely moving mice. We observed that GluN2 subunits differentiate between stimulus patterns, whereby GluNA is critically required for weaker and less persistent forms of LTP, and GluN2B is required for LTP that is very robust and persistent. These data indicate that the GluN2 subunits act as specific detectors for, and molecular transducers of, the nature and durability of synaptic plasticity.

## Materials and Methods

### Laboratory Animals

The experiments conducted were performed according to the European Communities Council Directive of September 22nd, 2010 (2010/63/EU) for care of laboratory animals with prior approval from the local ethics committee (Landesamt für Naturschutz, Umweltschutz und Verbraucherschutz, Nordrhein Westfalen). All measures were taken to minimize animal suffering and to reduce the number of animals.

Experiments were performed on male GluN2A knockout (KO) mice (Sakimura et al., 1995; Berberich et al., 2005), and their wild-type (WT) littermates. Mice were required to attain the minimum weight of 22 g before they underwent the surgical electrode implantation procedure. The animals were housed in vivariums (Scantainer Ventilated Cabinets, Scanbur A/S, Denmark) in which a constant temperature (22 ± 2°C) and humidity (55 ± 5%) was maintained. The housing environment had a constant 12 h light-dark cycle (lights on from 08:00 h to 20:00 h) and the animals had access to food and water *ad libitum*. After surgery, animals were housed individually and were allowed at least 7 days of recovery before the commencement of electrophysiological and pharmacological experiments. All surgical procedures and experiments were conducted during the day.

### Surgery

Mice were anesthetized using sodium pentobarbital (52 mg/kg, i.p.) before and during the electrode implantation procedure, as described previously (Buschler et al., 2012). Bipolar stimulating electrodes were implanted into the right Schaffer collateral pathway of the dorsal hippocampus (anterioposterior (AP): −2.0 mm; mediolateral (ML): 2.0 mm from bregma; dorsoventral (DV): ~1.4 mm from brain surface) and monopolar recording electrodes were implanted in the right ipsilateral CA1 *Stratum radiatum* (AP: −1.9; ML: 1.4; DV: ~1.2) to monitor the evoked potentials at the Schaffer Collateral-CA1 synapses. Test-pulse recordings during surgery aided depth adjustment of the electrodes. Electrophysiological recordings were performed in 20 (L) × 20 (W) × 30 (H) cm recording chambers, in which the mice could move freely and had access to food and water *ad libitum*. Animals were transferred from their home cages into the experiment room 1 day before the start of experiments to ensure adequate acclimatization to the environment, and were placed in the recording chambers in the evening before the experiment began.

### Measurement of Evoked Potentials

Each mouse had its socket connected via a swivel connector to the recording/stimulation system by means of wires suspended above the recording chamber. This enabled monitoring of evoked potentials while the animal freely behaved. The field excitatory postsynaptic potential (fEPSP) was analyzed by determining the maximal slope through the five steepest points obtained on the first negative deflection of the potential. To obtain these measurements, an evoked response was generated in the *Stratum radiatum* by stimulating the Schaffer collaterals with single biphasic square waves of 0.2 ms duration per half-wave, generated by a constant current isolation unit.

An input-output (IO) relationship was determined in the morning before each experiment. The largest obtainable fEPSP was found for every individual animal (maximum intensity used 125 μA) and the intensity that elicited 40% of the maximum fEPSP was used for test-pulse stimulation or induction of synaptic plasticity. Basal synaptic transmission was determined by applying test-pulse stimulation. For each time-point measured during the experiments, five test-pulses were applied at 40 s intervals and the fEPSP responses were averaged to represent one time-point. The first six time-points, which were recorded at 5 min intervals, were averaged and all time-points throughout the entire recording duration were expressed as a mean percentage (± standard error of the mean) of this value. Immediately after the 6th time-point, high-frequency stimulation (HFS) was applied to induce LTP. Where Ifenprodil was applied (see below), we first recorded the baseline for 30 min, then injected the NMDAR antagonist, then recorded baseline for a further 30 min and then applied HFS.

After HFS, three time-points were recorded at 5 min intervals, all subsequent recordings were made at 15 min intervals for 4 h post-HFS. A further 1 h of recordings were performed the next day, roughly 24 h after the experiment began to determine the degree of persistency of any changes in synaptic transmission.

Cortical electroencephalography (EEG) activity was monitored throughout the course of the experiment for the occurrence of seizure activity. No behavioral changes, or EEG activity, indicating seizures were observed. Postmortem histological analysis of the electrode localizations was performed for each animal to verify whether the electrodes were positioned in their respective desired positions as described before (Goh and Manahan-Vaughan, 2013a). Data from animals, in which electrode misplacements, or anatomical misconfigurations were found, were excluded from analysis.

### Induction of Synaptic Plasticity

Early-long term potentiation (E-LTP, <60 min) was induced using one train of HFS comprising 100 Hz (50 pulses). LTP that lasts for 2–4 h was induced by HFS at 100 Hz given as two trains of 50 pulses, separated by 5 min. Late LTP (L-LTP) that lasts at least 24 h was induced by HFS at 100 Hz given as *four* trains of 50 pulses (at 5 min intervals). We chose to use the term E-LTP (rather than short-term potentiation, STP) to describe potentiation that lasts for up to 60 min, as *in vitro* studies that examine LTP in hippocampal synapses, classify potentiation that lasts for ca. 60 min as LTP and not STP (Bliss and Collingridge, 1993; Bear and Malenka, 1994; Malenka and Bear, 2004). We chose to use different numbers of trains of 100 Hz to elicit synaptic potentiation of different durations based on prior experience with regard to synaptic plasticity protocols in freely behaving mice (Buschler et al., 2012).

### Compounds

The GluN2B-antagonist Ifenprodil (Gallagher et al., 1996; Williams, 2001) was applied as Ifenprodil (+)-tartrate salt (Sigma, Taufkirchen, Germany) and was dissolved in distilled water and applied at a dose of 10 mg/kg, i.p., in 10 ml/kg. An equivalent dose is known to disrupt spatial learning (Ma et al., 2011).

### Data Analysis

Experiments and analysis were conducted experimenter-blind. Data was first separated into the respective statistical cohorts at the end of the experimental series (WT vs. KO and Ifenprodil vs. vehicle). Electrophysiological data between groups was analyzed using two-way analysis of variance (ANOVA) with the repeated measures factor (TIME) and between-groups interaction factor (GROUP) being used to evaluate differences in plasticity between wild type and transgenic animals or comparisons of vehicle and antagonist application, or to assess if synaptic plasticity responses were significant compared to test-pulse stimulated controls. *Post hoc* Fisher LSD tests were used to assess for differences at specific time-points. The fEPSPs from the period after electrical HFS to the end of the experiment was compared between groups in order to assess the statistical difference in any change of synaptic strength. The significance level was set at *p* < 0.05.

## Results

### Basal Synaptic Transmission is Stable in The CA1 Region of Freely Behaving Wild-Type and GluN2A-KO Mice

**Figure 1:**
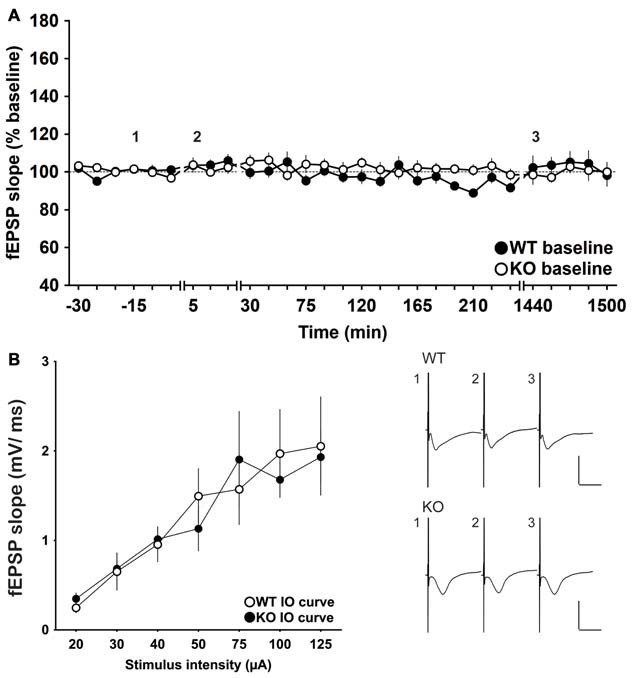
**Basal synaptic transmission and input/output (IO) properties are equivalent in freely behaving GluN2A-knockout (KO) mice compared to wildtypes. (A)** Field excitatory postsynaptic potentials (fEPSPs) evoked by test-pulse stimulation were stable and comparable in wild-type (WT) and GluN2A-KO mice over the 25 h monitoring period (“Baseline”). **(B)** IO properties were similar in WT and KO mice. The stimulus intensity was increased stepwise in the range of 20–125 μA. Insets: examples of fEPSPs evoked at the time-points indicated by the numbers in **(A)**. Horizontal scale bar: 10 ms, vertical scale bar: 2 mV.

To assess basal synaptic transmission and stability of the potentials test-pulse evoked responses and IO curves were recorded and compared in WT and GluN2A KO mice (Figure 1). No significant differences were found between fEPSPs that were evoked by test-pulse stimulation of WT (*n* = 10), compared to KO mice (*n* = 11, ANOVA: *F*_(1,19)_ = 0.512, *p* = 0.48; Figure 1A). Similarly IO curves (Figure 1B), that examined the stimulus-response relationships of fEPSPs evoked by stepwise increases in stimulus intensity, showed no significant differences between WT and KO mice (*n* = 8 each; ANOVA *p* > 0.05).

### Early-LTP is Impaired in GluN2A-KO Mice

To assess the involvement of GluN2A in LTP, different high frequency stimulation (HFS) protocols were used, based on prior experience as to the most effective protocols for eliciting LTP of different magnitude and persistencies in freely behaving mice (Buschler et al., 2012). We restricted the stimulation frequency used to 100 Hz, because lower HFS frequencies and theta-burst stimulation (TBS) do not induce LTP in the CA1 region of freely behaving mice (Buschler et al., 2012), whereas higher frequencies trigger epileptiform seizures in the CA1 region *in vivo*.

**Figure 2:**
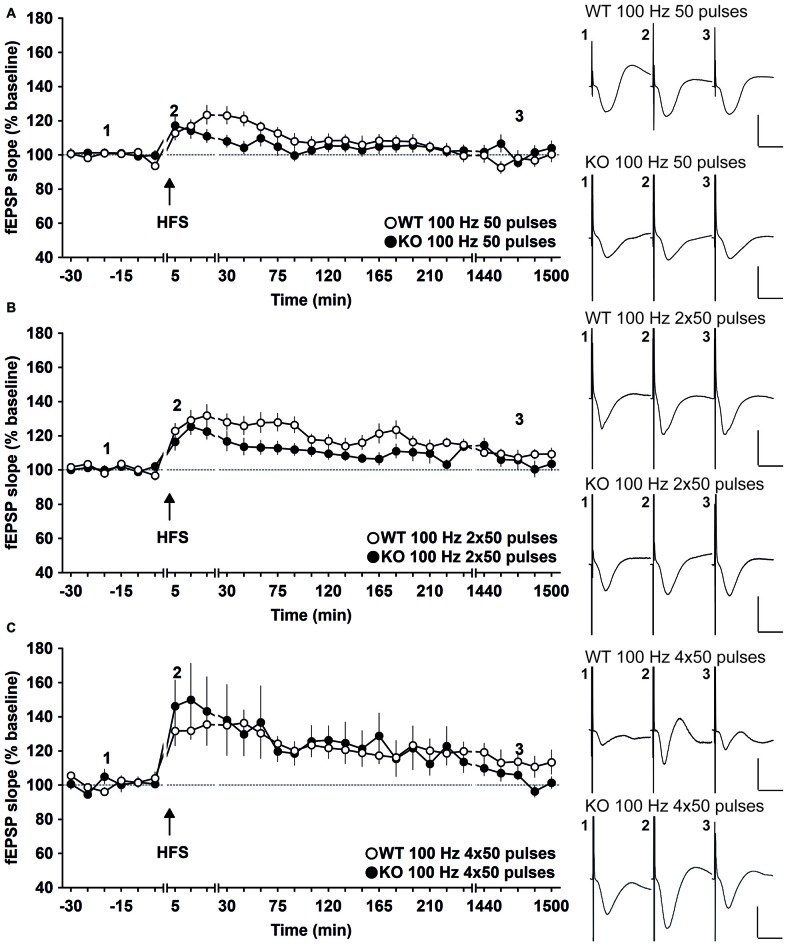
**Early-long-term potentiation (E-LTP) is impaired, whereas LTP and late-LTP (L-LTP) are unaltered in freely behaving GluN2A-KO mice. (A)** High-frequency stimulation (HFS) comprising 50 pulses at 100 Hz evokes significant E-LTP (<1 h) in WT mice. The same protocol, when given to GluN2A-KO mice, results in a significant impairment of E-LTP. **(B)** HFS comprising 100 Hz, given as two bursts of 50 pulses, induces robust LTP (2–4 h) in both WT and KO mice. **(C)** A stronger HFS that comprises 100 Hz, given as four bursts of 50 pulses induces L-LTP, (>24 h) in WT mice. KO animals respond to this protocol with L-LTP that is not significantly different to WT L-LTP. Insets: examples of fEPSPs evoked at the time-points indicated by the numbers. Horizontal scale bar: 10 ms, vertical scale bar: 2 mV.

First, we examined the involvement of GluN2A in E-LTP in behaving mice. Here, we stimulated the Schaffer collaterals with HFS at 100 Hz given as a single 50 pulse train. In WT mice (*n* = 12), this protocol induced significant E-LTP that lasted for about 1 h (Figure 2A; ANOVA: *F*_(1,20)_ = 8.297, *p* < 0.01; interaction effect *F*_(22,440)_ = 3.237, *p* < 0.001, compared to test-pulse stimulated WT controls, *n* = 10). GluN2A-KO mice (KO, *n* = 13) exhibited a significantly impaired response to this HFS protocol (Figure 2A; ANOVA: *F*_(1,22)_ = 0.913, *p* = 0.35; interaction effect *F*_(22,484)_ = 1.480, *p* = 0.07, compared to test-pulse stimulated KO mice, *n* = 11). Furthermore, statistical analysis of the initial 45 min after HFS revealed a significantly higher potentiation in the WT mice (*n* = 12) compared to the KO mice (*n* = 13; ANOVA: *F*_(1,23)_ = 5.427, *p* = 0.0289; interaction effect *F*_(4,92)_ = 1.734, *p* = 0.15).

### LTP (2–4 h) is Unaffected in GluN2A-KO Mice

Given the impairment of E-LTP in the GluN2A-KO mice, we explored whether GluN2A is also required for a more robust form of hippocampal LTP that persists for 2–4 h in freely behaving mice. Here, we applied 100 Hz HFS in two 50 pulse trains given 5 min apart (Figure 2B). In WT mice (*n* = 15) this protocol induced LTP (>2 h; Figure 2B; ANOVA: *F*_(1,23)_ = 34.101, *p* < 0.001; interaction effect *F*_(17,391)_ = 0.725, *p* = 0.78, compared to test-pulse stimulated WT controls, *n* = 10). KO mice (*n* = 17) expressed LTP that was also significant from test-pulse stimulated KO controls (*n* = 11; ANOVA: *F*_(1,26)_ = 5.320, *p* = 0.0293; interaction effect *F*_(22,572)_ = 1.614, *p* = 0.0382; Figure 2B).

Although a tendency towards slightly weaker potentiation values was evident 45–90 min after HFS, the overall profile and persistence of LTP was not significantly different in WT (*n* = 15) and KO animals (*n* = 17; ANOVA: *F*_(1,30)_ = 3.943, *p* = 0.0562; interaction effect *F*_(22,660)_ = 1.092, *p* = 0.35).

### L-LTP (≥24 h) is Unaffected in GluN2A-KO Mice

In freely moving mice, L-LTP that persists for over 24 h can be induced in the CA1 region by means of repetitive afferent stimulation at 100 Hz (Buschler et al., 2012). We also assessed if GluN2A is required for this form of LTP. For this, we stimulated afferents with 100 Hz HFS, given as four 50 pulse trains given 5 min apart (Figure 2C), WT mice (*n* = 12) responded to this protocol with L-LTP (≥24 h; ANOVA: *F*_(1,20)_ = 32.156, *p* < 0.0001; interaction effect *F*_(22,440)_ = 1.573, *p* < 0.05, compared to test-pulse stimulated WT controls, *n* = 10). KO animals (*n* = 11) also expressed significant L-LTP (≥24 h; ANOVA: *F*_(1,20)_ = 4.8786, *p* = 0.039; interaction effect *F*_(22,440)_ = 2.3607, *p* < 0.001, compared to responses evoked in test-pulse stimulated KO controls *n* = 11). No significant difference in the profile of L-LTP was evident in WT (*n* = 12) and KO (*n* = 11) animals (ANOVA: *F*_(1,21)_ = 0, *p* = 0.9979; interaction effect *F*_(22,462)_ = 0.7588, *p* = 0.7769).

### Antagonism of GluN2B Has no Effect on E-LTP But Prevents The Establishment of L-LTP in Wild-Type Mice and in GluN2A-KO Mice

Differences in the relative dependency of LTP on GluN2 subunits of the NMDAR have been reported *in vitro* (Köhr et al., 2003; Liu et al., 2004; Massey et al., 2004; Berberich et al., 2005) and in pharmacological studies in anesthetized rats *in vivo* (Fox et al., 2006). Here, we assessed if pharmacological antagonism of GluN2B affects LTP in the CA1 region of freely behaving mice.

**Figure 3:**
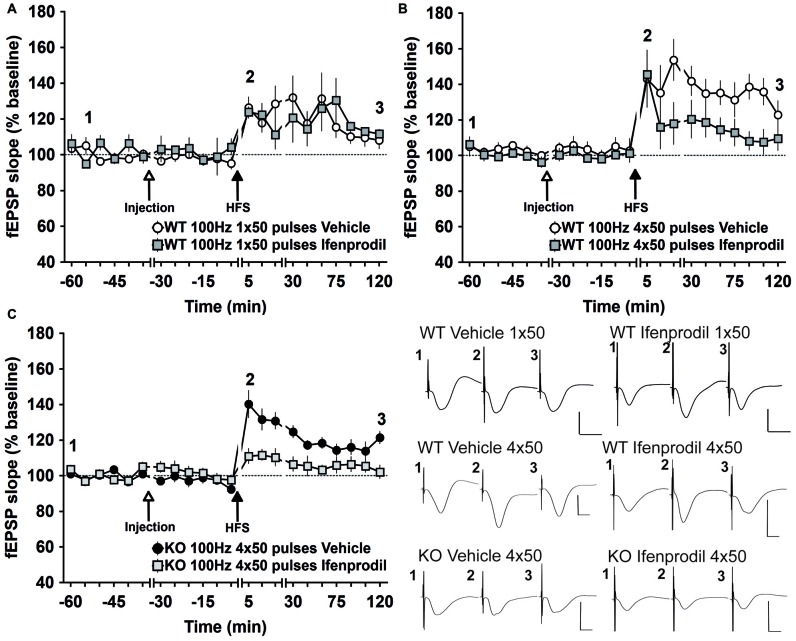
**Antagonism of GluN2B in freely behaving WT mice prevents LTP (2 h) but not E-LTP (<1 h), antagonism of GluN2B in GluN2A-KO mice prevents LTP (>2 h). (A)** Specific antagonism of GluN2B by Ifenprodil has no effect on E-LTP induced in WT mice.** (B)** Ifenprodil elicits no effect on E-LTP induced in WT, whereas LTP (2 h) is prevented.** (C)** Ifenprodil prevents E-LTP and LTP in GluN2A KO mice. Insets: examples of fEPSPs evoked at the time-points indicated by the numbers. Horizontal scale bar: 10 ms, vertical scale bar: 2 mV.

First, we assessed effects on E-LTP (<1 h): HFS (50 pulses) given in the presence of the GluN2B antagonist, Ifenprodil (10 mg/kg, i.p.), resulted in E-LTP (*n* = 5) that was not significantly different to E-LTP induced in vehicle-treated WT mice (*n* = 8; Figure 3A; ANOVA: *F*_(1,11)_ = 0.9166, *p* = 0.3589; interaction effect *F*_(9,99)_ = 0.8352, *p* = 0.5854).

Then we examined the effect of applying HFS as 4 × 50 pulse trains in the presence of Ifenprodil. This protocol results in L-LTP (Figure 2C). Here, we observed that in WT mice, treatment with the GluN2B antagonist, Ifenprodil (*n* = 6), resulted in E-LTP that was equivalent to that seen in vehicle-treated controls (*n* = 6; ANOVA, *t* = 5 min until *t* = 45 min post-HFS: *F*_(1,8)_ = 0.2406, *p* = 0.6370; interaction effect *F*_(4,32)_ = 0.4193, *p* = 0.7935; Figure 3B). Subsequent LTP was significantly prevented however (Figure 3B; ANOVA, *t* = 60 min until *t* = 120 min post-HFS: *F*_(1,10)_ = 5.9693, *p* = 0.0347; interaction effect *F*_(4,40)_ = 1.6078, *p* = 0.1912).

In GluN2A-KO mice, treatment with Ifenprodil (*n* = 8), resulted in a significant impairment of all phases of LTP, including E-LTP (Figure 3C) compared to vehicle-treated KO controls (*n* = 8; ANOVA, *t* = 5 min until *t* = 45 min post-HFS: *F*_(1,14)_ = 15.256, *p* = 0.0016; interaction effect *F*_(9,126)_ = 1.763, *p* = 0.0816).

These data indicate that whereas GluN2A signaling is required for the successful induction of weak forms of synaptic plasticity, such as E-LTP, GluN2B signaling is required for the induction of more persistent forms of LTP.

## Discussion

The results of this study demonstrate that in the behaving mouse, the NMDAR GluN2A and GluN2B subunits act as sensors that discriminate between patterns of incoming afferent stimuli, and depending on their activity, drive the encoding of this information in the form of transient or persistent synaptic plasticity. Whereas GluN2A mediates the induction of weaker and less persistent forms of LTP, GluN2B enables the induction of LTP that is very robust and persistent. These distinct signaling properties are pivotal to the subjective discrimination of synaptic information encoding, and can be expected to support the segregation of experience into synaptic, and perhaps cognitive, memories of different qualitative significance.

Although in the CA1 region *in vitro*, LTP can be induced with afferent stimulation frequencies as low as 25 Hz, and as high as 200 Hz (Grover and Teyler, 1990), in freely behaving mice, the frequency spectrum with which LTP can be induced, is very small. Frequencies below (Buschler et al., 2012), or above 100 Hz (Goh and Manahan-Vaughan, 2013a) are completely ineffective. The optimal frequency for the induction of CA1 LTP is 100 Hz, and strikingly, it is indeed the *pattern* of stimuli at 100 Hz that determines the persistency of LTP. This finding aligns with the suggestion by others, that particular patterns of afferent stimulation in the 100 Hz frequency range may emulate spike discharge patterns of hippocampal neurons that occur during information processing (Larson et al., 1986).

It has been proposed that the pattern of activity of NMDARs determines whether LTP or LTD are expressed (Malenka and Bear, 2004). Our data provide the first evidence that this is the case in the *behaving* animal. The contradiction by our findings, of data produced through pharmacological studies conducted using the hippocampal slice preparation (Volianskis et al., 2013) or in rats *in vivo* (Ge et al., 2010), may derive from the dearth of specific antagonists for GluN2A. The current ligand of choice is NVP-AAM077, but it exhibits only a 10-fold preference for GluN2A over GluN2B (Feng et al., 2004; Paoletti and Neyton, 2007). Differences, from results obtained *in vitro*, in transgenic mice that lack GluN2A or GluN2B may not only derive from the fact that the stimulation protocols used to elicit synaptic plasticity *in vitro*, do not correspond to those that are effective *in vivo* (see above), but also from the fact that in the behaving animal, homeostatic regulation of the subunit composition of the NMDAR occurs (Barria and Malinow, 2002; Ward et al., 2006; Matta et al., 2011), that is driven by the prior experience of the synapse (Xu et al., 2009). These effects can be extremely rapid, localized to individual synapses, and potently influence the propensity of the synapse to express synaptic plasticity (Lee et al., 2010). In the hippocampus of the behaving animal it is not unreasonable to assume that the experience-dependent, subcellular subunit composition of the NMDAR is different to that of the hippocampal slice.

Our data indicate that when afferent activity is transient (e.g., one train of 100 Hz stimulation), E-LTP is enabled by means of GluN2A, but when afferent activity comprises a repetitive pattern, albeit at the same afferent frequency as that used to evoke E-LTP, LTP that increasingly depends on GluN2B is induced. The difference in dependency of E-LTP in GluN2A and LTP on GluN2B align with reports from *in vitro* studies that GluN2A may respond to weaker stimuli compared to GluN2B (Köhr et al., 2003; Berberich et al., 2005, 2007), has a higher opening probability in response to glutamate (Erreger et al., 2005), and activates faster (Vicini et al., 1998). This may explain why this subunit is specifically involved in the transduction of weak afferent stimuli into E-LTP. The support by GluN2B of more potent and persistent forms of LTP can be explained by its specific kinetics. Although GluN2B may require a more intense postsynaptic depolarization to remove the voltage-dependent Mg^2+^ block of the NMDAR (Erreger et al., 2005; Clarke et al., 2013), when activated, GluN2B-containing NMDAR support twice as much charge transfer (as GluN1/GluN2A-receptors), deactivate slower and support a greater Ca^2+^ influx per unit of current (Vicini et al., 1998; Sobczyk et al., 2005). Ca^2+^ influx is a major determinant of the degree, duration and direction of change of synaptic strength that results from patterned afferent stimulation. Controlling the amount of postsynaptic Ca^2+^ entry through NMDARs by modulating the relative degree of NMDAR antagonism during tetanic stimulation (that typically results in CA1 LTP in the absence of the antagonist) results in LTD, or no change in synaptic strength with lower and higher antagonist concentrations, respectively (Cummings et al., 1996). Furthermore, very robust hippocampal LTP that is induced by strong tetanic stimulation in freely behaving rats becomes curtailed in its magnitude if the tetanus is applied in the presence of AP5 (Manahan-Vaughan et al., 1998) an NMDAR antagonist that acts via binding to the GluN2B subunit (Laube et al., 1997).

To reconcile the dichotomous GluN2A- vs. GluN2B-signaling with the existence of triheteromeric NMDARs (Gray et al., 2011; Rauner and Köhr, 2011; Tovar et al., 2013), the downstream activation of GluN2A- vs. GluN2B-associated signaling molecules needs to be considered. The intracellular coupling of GluN2B indicates that it may play a specific role in the support of persistent, protein-synthesis-dependent, forms of LTP. It exhibits high affinity binding to Calcium/Calmodulin-dependent kinase II (CaMKII; Strack and Colbran, 1998) and anchors CaMKII in its active form to the synapse (Bayer et al., 2001). This property is a critical component in the expression of robust LTP, and it is not supported by GluN2A (Barria and Malinow, 2005). By contrast, GluN2A may support LTP by activation of the Ras/Erk Mitogen-activated protein (MAP) kinase pathway (Jin and Feig, 2010). Thus, two complementary intracellular pathways, mediated by GluN2A and GluN2B, may support E-LTP and persistent LTP (≥4 h), respectively. Interestingly, the specific regulation by GluN2B of CaMKII suggests that this subunit may be predominantly involved in the support of LTP and not LTD, and experimental evidence supports this possibility (Zhou et al., 2007). Furthermore, in freely behaving mice that exhibit persistent autophosphorylation of CaMKII, L-LTP (elicited with 100 Hz) is potently impaired (Goh and Manahan-Vaughan, 2015).

The pattern-specific activation of GluN2A and GluN2B signaling may relate to their role in hippocampus-dependent memory. Hippocampal encoding of long-term memory can be differentiated into memories that are short- or long-term, occur through a single experience or arise through cumulative learning (Olton and Samuelson, 1976; Misane et al., 2005; Antunes and Biala, 2012). Although it is assumed that cumulative learning can enable a short-term memory to be consolidated into long-term memory, the formation of long-term memory can also bypass interim short-term storage (Izquierdo et al., 1999). Striking, in light of our observation that GluN2B is needed for persistent LTP (≥4 h), is the finding that GluN2B is required for context-dependent fear-conditioning (Wang et al., 2009). This form of one-trial learning is extremely robust (Misane et al., 2005), is encoded by LTP in the CA1 region (Whitlock et al., 2006), and can be expected to be triggered by a convergence of multiple afferent inputs from strongly activated brain regions (Maren et al., 2013). Given its requirement for E-LTP, GluN2A may be particularly involved in short-term memory, and behavioral studies using the same transgenic strain as was used here, confirm that this may indeed be the case (Bannerman et al., 2008). But LTD that is associated with novel object-place learning, and may not depend on GluN2B (Goh and Manahan-Vaughan, 2015), is persistent and protein-synthesis-dependent (Kemp and Manahan-Vaughan, 2013). Although some behavioral studies in transgenic animals have not identified a clear delineation of specific memory subforms with specific GluN2 subtypes (Shipton and Paulsen, 2013), a recent study indicated the specific involvement of GluN2A in spatial pattern separation, that is believed to be processed by the dentate gyrus, but not in temporal pattern separation, that is believed to be processed by the CA1 region (Kannangara et al., 2014). Hippocampus-dependent memory forms, such as spatial memory, are tightly associated with both LTP and LTD (Kemp and Manahan-Vaughan, 2004, 2007), and robust synaptic plasticity can be triggered by a single experience (Manahan-Vaughan and Braunewell, 1999; Goh and Manahan-Vaughan, 2013b), or develop through cumulative learning (Uzakov et al., 2005). Furthermore, a subregional differentiation in the encoding of different aspects of spatial content, though synaptic plasticity has been reported (Kemp and Manahan-Vaughan, 2007). It is therefore, likely that at the level of hippocampus-dependent memory, both GluN2 subtypes are involved: with the assumption that synaptic plasticity encodes memory, it can be expected that, through the fine-tuning of the direction of change of synaptic strength, the determination of the magnitude of change, and the persistency of the plasticity response, the GluN2 subtypes play a central role in hippocampal encoding of distinct components of memory.

In summary, our data show, in freely behaving mice, that whereas GluN2A is required for transient forms of plasticity, GluN2B is required for L-LTP. We propose that incoming afferent stimuli, that can be expected to vary in their intensity and duration, based on the saliency and content of the experience, determine the relative activation of GluN2A and GluN2B. This can serve to prioritize short-term or long-term information encoding, whereby the relative interplay of subunit activation may also determine the degree of encoding by LTP and LTD. In effect, it may not be the afferent frequency *per se*, but rather the pattern with which afferent information reaches the hippocampus, that determines the relative activation of GluN2A and GluN2B, and thereby the durability and the precise direction of change of synaptic strength.

## Author Contributions

The concept and strategy for the study were developed by DM-V and GK; JJB and AB conducted the experiments and data analysis. Data interpretation was conducted jointly by all authors. DM-V and GK wrote the article with contributions from AB and JJB.

## Conflict of Interest Statement

The authors declare that the research was conducted in the absence of any commercial or financial relationships that could be construed as a potential conflict of interest.
